# Impact of proprioception on the perceived size and distance of external objects in a virtual action task

**DOI:** 10.3758/s13423-021-01915-y

**Published:** 2021-03-29

**Authors:** Wladimir Kirsch, Wilfried Kunde, Oliver Herbort

**Affiliations:** 1 Department of Psychology, University of Würzburg, Würzburg, Germany; 2 Institut für Psychologie III der Universität Würzburg, Röntgenring 11, D-97070 Würzburg, Germany

**Keywords:** Visual perception, Motor control

## Abstract

Previous research has revealed changes in the perception of objects due to changes of object-oriented actions. In present study, we varied the arm and finger postures in the context of a virtual reaching and grasping task and tested whether this manipulation can simultaneously affect the perceived size and distance of external objects. Participants manually controlled visual cursors, aiming at reaching and enclosing a distant target object, and judged the size and distance of this object. We observed that a visual–proprioceptive discrepancy introduced during the reaching part of the action simultaneously affected the judgments of target distance and of target size (Experiment 1). A related variation applied to the grasping part of the action affected the judgments of size, but not of distance of the target (Experiment 2). These results indicate that perceptual effects observed in the context of actions can directly arise through sensory integration of multimodal redundant signals and indirectly through perceptual constancy mechanisms.

**Supplementary Information:**

The online version contains supplementary material available at 10.3758/s13423-021-01915-y.

Changes in visual perception have been reported under diverse conditions in which certain characteristics of the body or of its action varied (for reviews, see Harris et al., 2015; Hommel et al., 2001; Proffitt, 2006; Proffitt & Linkenauger, 2013; Witt, 2011a; Zwickel & Prinz, 2012). These “action specific effects” have been often observed when body and manipulated objects were not in direct contact, such as in tool use, although their origin is not well understood (Firestone & Scholl, 2016; Philbeck & Witt, 2015). We recently noted that these effects resemble well-known interactions between senses, such as between vision and touch, and might thus obey the same known principles of sensory integration of multimodal signals even though the signals are spatially separated (Kirsch et al., 2017; Kirsch & Kunde, 2019a, 2019b; see also Debats et al., 2017a, 2017b; Debats & Heuer, 2018a, 2018b).

Consider that haptic signals can provide information about the same object as visual signals, such as in natural grasping. Importantly, when a discrepancy between both signals is introduced, the haptic signal (e.g., current hand opening) attracts the visually perceived size of the grasped object (Ernst & Banks, 2002). This and similar effects indicate that the object’s percept combines both haptic and visual signals (e.g., Helbig & Ernst, 2007). Note that such a crossmodal discrepancy or conflict is omnipresent in the studies on action-specific perception, which typically vary a body-related variable and hold the visual input constant. Consider also that, in theory, body-related signals can inform observers about object features in many interactions with the environment because body features often covary with environmental features. In throwing an object, for example, features of object motion, such as trajectory or final distance, are strongly correlated with body features associated with setting the object in motion, such as with magnitude and direction of applied forces. Thus, perceptual changes reported in several settings in the context of actions can be considered as outcomes of sensory integration of redundant multimodal signals.

In a series of previous studies, we used a virtual grasping task in which participants repeatedly enclosed visual objects by manually controlled visual cursors and measured the perceived objects’ size and hand opening (Kirsch et al., 2017; Kirsch & Kunde, 2019a, 2019b). We introduced a discrepancy between the visual object size and hand opening (i.e., visual–proprioceptive conflict) and observed mutual attraction biases between proprioceptive and visual signals that varied with relative reliability of visual information. These results confirmed main predictions of the sensory integration approach. An important aspect of these findings is that, unlike the natural grasping, indicators of sensory integration were observed in the absence of direct contact between the body and a distant object (i.e., under conditions conceptually similar to situations in which action specific effects have been frequently reported).

In the present study, we further explored this approach. In particular, we tested whether visual–proprioceptive discrepancies implemented in a virtual reaching and grasping task influence size constancy mechanisms, which ensure the same perception of object size, irrespective of object’s distance. A formal description of size constancy is known as the size–distance invariance hypothesis (SDIH). According to the SDIH, the perceived size of an object (oriented uprightly to the line of sight) is computed by the multiplication of a function of the retinal size with perceived distance: Perceived size = tan (retinal image size) × perceived distance (e.g., Epstein et al., 1961; Kaufman et al., 2006). If proprioception informs vision about distant objects in the context of goal-directed actions, as we suggested, then an impact of visual–proprioceptive discrepancies on the perception of distance should also be expressed in perceived size and vice versa; an impact on perceived size could propagate to the perception of distance (see also, e.g., Sperandio & Chouinard, 2015).

Resolving this issue could help us to better understand the origin of previous observations and to predict new phenomena. For example, a number of studies indicated changes in size perception associated with changes in action ability (e.g., Cañal-Bruland & van der Kamp, 2009; Cooper et al., 2012; Gray et al., 2014; Kirsch et al., 2014; Lee et al., 2012; Wesp et al., 2004; Witt et al., 2008; Witt & Dorsch, 2009; Witt & Proffitt, 2005). Increased ability to hit a golf hole, for example, was associated with larger judgments of object size (e.g., Witt et al., 2008). Moreover, action ability proved to affect the perceived distance in another group of studies (Kirsch & Kunde, 2013a, 2013b; Linkenauger et al., 2015; Witt, 2011b; Witt et al., 2005; Witt & Proffitt, 2008). For example, the length of a virtual arm affected the perceived distance to a target object after reaching experience: A longer arm (i.e., a larger reaching ability) decreased the distance estimates as compared with a shorter arm (Linkenauger et al., 2015). Both observations could originate from changes in distance perception or, on the other hand, from changes in size perception. Moreover, changes in size perception observed in the first group of studies should be accompanied by changes in distance perception and, vice versa, changes in distance perception observed in the second group of studies should be accompanied by changes in size perception. Some indirect evidence for such an effect transfer already exists (Suh & Abrams, 2018; see also Stefanucci & Proffitt, 2009; Stefanucci & Storbeck, 2009; for related observations in the context of emotions).

Below, we report two experiments, in which participants virtually reached and grasped a distant object and judged the distance and size of that object. The rationale was as follows. The cue integration approach suggests that visual and proprioceptive signals are integrated to the extent they provide information about the same object feature (Deroy et al., 2016; Ernst, 2006; Shams & Beierholm, 2010). For example, reaching for an object informs one about the distance of the object, but usually not about its size. Grasping an object, in contrast, informs one about the object size, but not about its distance. Accordingly, the reaching component of a goal-directed movement (“movement distance” hereafter) should serve as a cue for distance perception and thus directly affect the perceived distance of the object, but not its size. The grasping component (“finger aperture” hereafter), in contrast, should serve as a cue for size perception and thus directly affect the perceived size of the object, but not its perceived distance. We varied visual–proprioceptive discrepancy created for either movement distance (Experiment 1) or finger aperture (Experiment 2) and explored whether these predicted effects on perceived object distance and size propagate to the other perceptual dimension according to the size constancy. To anticipate the results, this was the case for Experiment 1, but not for Experiment 2.

## Experiment 1

**Fig. 1:**
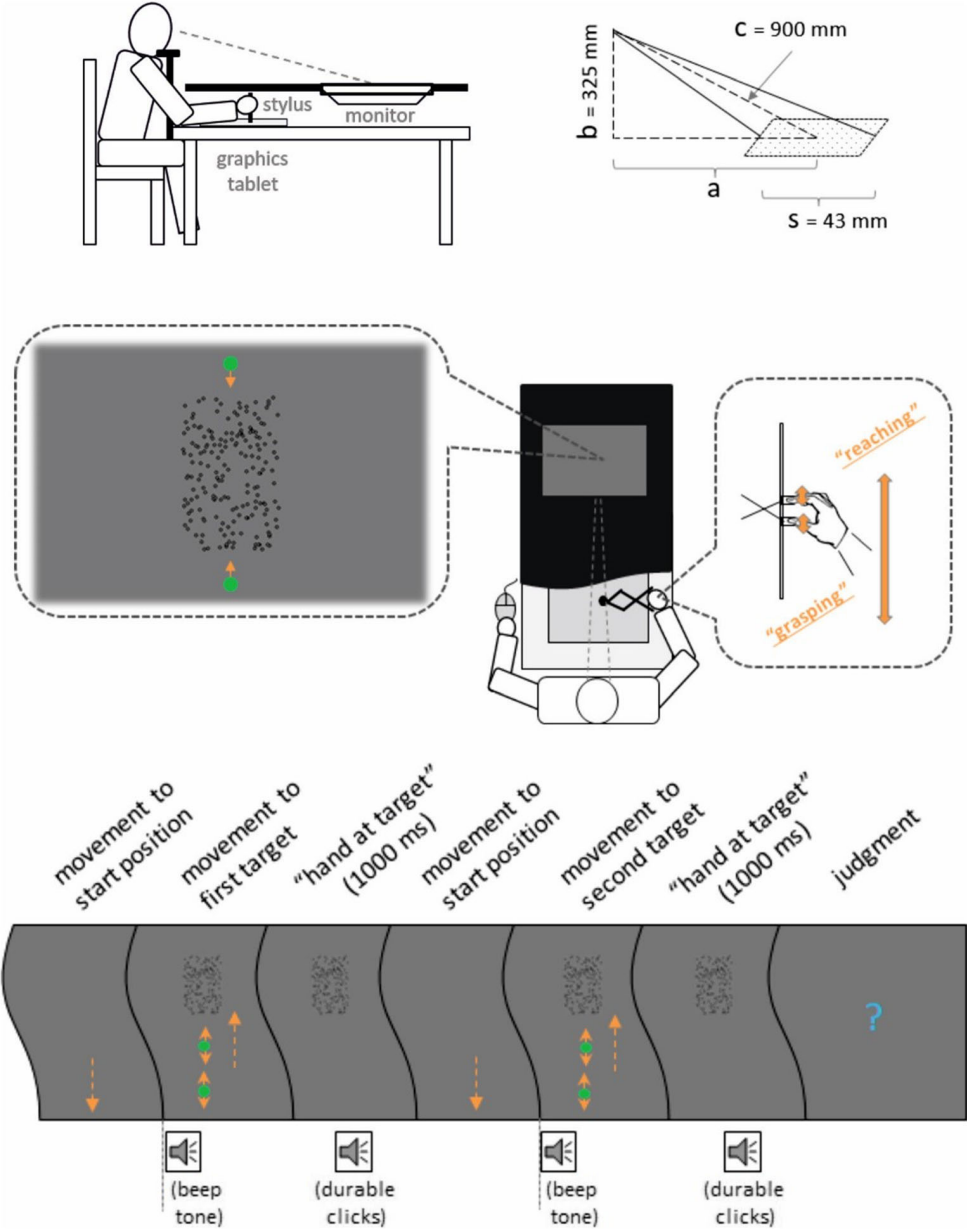
Experimental setup (upper and middle parts) and main trial events (lower part) in Experiments 1 and 2. The letters (a, b, c, and s) shown in the right upper part describe the physical relation across the observer’s viewpoint, the location, and the size of the standard stimulus. Stimuli are not drawn to scale

Participants manually controlled a pair of visual cursors aiming at reaching and enclosing a distant rectangular target object (see Fig. 1). We varied the gain of the reaching component of the hand movement (i.e., the transformation of the hand movement distance into the cursor movement distance) and measured the perception of the target distance and size. Previous research suggested that an increase in hand movement distance should increase the perceived distance to the target under the present conditions. For example, increasing the arm’s reach by enlarging the arm avatar in a virtual environment proved to decrease the distance to a target object being reached (Linkenauger et al., 2015; also, see the Introduction and Kirsch & Kunde, 2015, for similar observations). According to the sensory integration approach, this and related effects arise because in reaching a given target object, the combined multimodal estimate of the final hand position, and thus of the position of the target, is closer to the participant’s body when the arm is virtually extended and movement distance decreases. In addition, an increase in hand movement distance was also expected to increase the perceived size of the target according to the SDIH (cf. also Sperandio & Chouinard, 2015).

### Methods

#### Participants

The sample size was estimated based on the data of the first five participants that served as a pilot data set. This initial analysis revealed effect sizes of *dz* = 0.5 and *dz* = 1.1 for the effects of hand movement distance on target size and distance, respectively. These values required 26 and seven participants (based on power of 1 − *β* = 0.80). Based on this estimate, we recruited 34 right-handed participants. Three of them took part in only one of two sessions. The data of another participant was unusable due to technical reasons. These participants were excluded prior to analyses. The final sample included 20 females and 10 males (*M*_age_ = 26 years, *SD* = 4 years). All participants had normal or corrected-to-normal vision. They gave their written informed consent for the procedures and received monetary compensation or course credit for their participation.

**Fig. 2:**
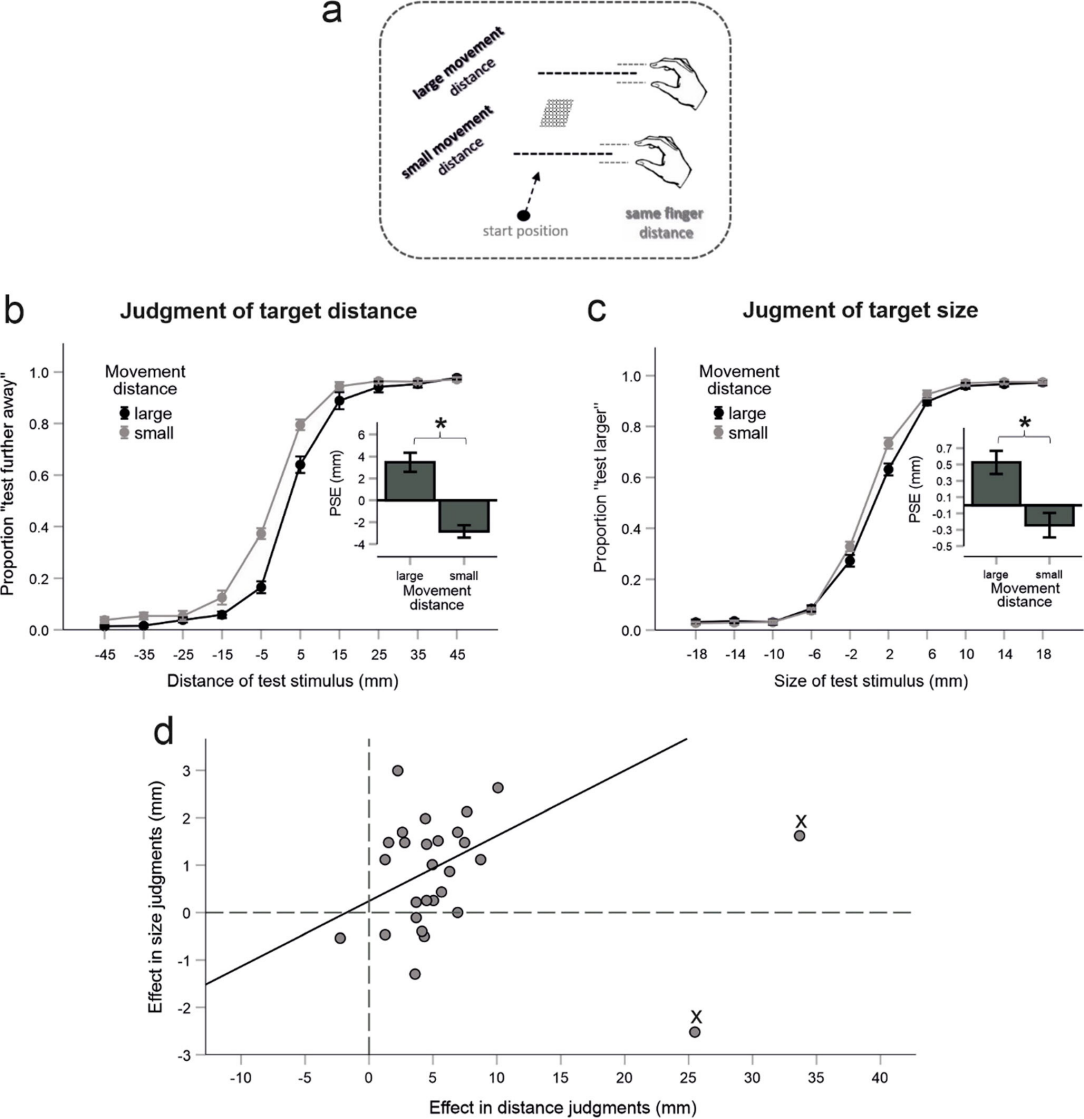
Experiment 1. **a** The critical variation of movement distance. **b** Results for the distance judgments. Shown are proportions of trials in which the test stimulus was judged as farther away as a function of movement distance and the distance of the test stimulus. Negative/positive values mean that the test stimulus was closer/farther away than the standard stimulus. Superimposed are the mean PSE values for the large and small movement distance conditions. **c** Results for the size judgments. Proportions of trials in which the test stimulus was judged as larger as a function of movement distance and the size of the test stimulus. Negative/positive values mean that the test stimulus was smaller/larger than the standard stimulus. Superimposed are the mean PSE values for the large and small movement distance conditions. **d** The effect of movement distance (large minus small) in the size judgments against the effect of movement distance in the target distance judgments for each participant. Crosses indicate participants who were not included in the correlation analysis. Error bars are standard errors indicating the variability across participants. Asterisks denote statistical significance (*p* < .05)

As estimation of effect sizes from small sample sizes could be inaccurate, we also computed confidence intervals (80%) for the expected effects based on the pilot data set (see also, e.g., Cocks & Torgerson, 2013; Lakens, 2021). These values amounted to 0.56 ± .76 mm for the size effect and 6.46 ± 4.14 mm for the distance effect. The effects observed in the whole sample of participants corresponded, on average, rather well to the means of the pilot data set and were thus well within their confidence intervals (see Fig. 2). Moreover, the predicted and observed effect sizes were quite similar (size: *dz* = 0.5 vs. *dz* = 0.6; distance: *dz* = 1.1 vs. *dz* = 0.9). Thus, the pilot sample rather accurately predicted the size and the magnitude of the effects of interest under the present conditions.

The study was conducted in accordance with the ethical guidelines (2016) of the German Psychological Society (DGPs) and has been approved by the local ethics committee (Ethikkommission des Institutes für Psychologie der Humanwissenschaftlichen Fakultät der Julius-Maximilians-Universität Würzburg, GZ 2019-04).

#### Apparatus

The experiment was performed in a dark experimental room. Stimuli were presented on a 19-in. monitor (Fujitsu Siemens P19-1; 1,280 × 1,024 pixels; 1 pixel = 0.294 mm; 60 Hz) that was placed horizontally in front of the participants, whose heads were supported by a chin rest (see the upper part of Fig. 1). The height of the chin rest was fix (about 20 cm above the level of the monitor) so that the distance between the eyes and the center of the monitor was about 90 cm. Participants used their right hand to manipulate a movement device that allowed recording of hand and finger movements. The movement device was mainly composed of a graphics tablet (Intuos 4 A4, Wacom), a digitizing stylus and a pincer-like construction (LEGO building blocks) that held the stylus up on the tablet and was moveable along a track (see the middle part of Fig. 1). Participants placed their fingers on two U-shaped plastic plates, which were fixed to the pincer construction and were thus mirror-symmetrically interlocked. The index and the middle fingers were bound together and placed on one plate, the thumb on the other. Moving the fingers together/apart moved the stylus to the left/right. Moving the hand forward/backward moved the stylus forward/backward. By recording the position of the stylus on the tablet, we thus were able to extract information about hand and finger movements. The vision of the hand during the movements (as well as of the movement device) was prevented by using a black cover positioned above the tablet. Perceptual judgments were made by pressing buttons of a computer mouse with the left hand. Auditory stimuli were presented through headphones.

#### Stimuli and trial procedure

The lower part of Fig. 1 illustrates the main trial events. At the beginning of each trial participants moved their fingers together and placed their hand at a start position (the location of a mechanical stop close to participants’ body). During this movement, the background of the display was always gray. After the start position was reached a short beep tone was presented, and a pair of green cursors (dots of 3 mm in size) and a first rectangular target object appeared at the lower and upper part of the display respectively. The target object was composed of a number of black unfilled circles (1.2 mm in diameter; density ~ 12 dots per cm^2^) randomly distributed along the rectangle. The width of the rectangle was always 3.1 cm. Its height and its location varied (see Design). Participants had to enclose the target object by the cursors (i.e., to virtually reach and grasp the object), and thus had to move their hand forward and the fingers apart until the cursors reach the edges of the object. A durable clicking noise was presented and the cursors disappeared when the correct hand and finger postures were adopted. Participants were asked to maintain this body state for 1 s and to perform corrective movements when the cursors left the edges of the target (i.e., when the hand location or the finger aperture changed). Then, the target object disappeared and participants had to move the hand back to the start position. After the start position was reached, a beep tone, the cursors and a second target object were presented. The second target object has to be virtually reached and grasped in the same way as the first target object. This movement was now followed by a blue question mark (5 mm), in response to which a perceptual judgment about the size or the distance of the target objects was made. When a judgment was made before the question mark appeared or when the finger or movement distance changed before the judgment was made an error display was presented and the trial was repeated.

#### Design

To measure size and distance perception we used a method of constant stimuli. One of the target objects served as a standard stimulus, another target object served as a test stimulus. The critical experimental variation was related to the standard stimulus. In particular, we varied the transformation of the hand movement distance to the cursor movement distance (i.e., gain) so that the same target object (4.3 × 3.1 cm, middle of the display) was reached either by a rather small hand movement (7 cm; “small movement distance”) or by a rather large hand movement (15 cm; “large movement distance”; see also Fig. 2a). Movements to the target object that served as a test stimulus were not transformed—that is, the distances of the hand movements corresponded to the distances covered by the cursors displayed on the monitor (and were 11 cm for the targets presented in the middle of the display).

There were two types of blocks of trials, “distance judgment blocks” and “size judgment blocks”, which differed in the characteristics of the test stimulus and in the required judgment. In the *distance judgment blocks,* the target object was always of the same size (4.3 × 3.1 cm). Its distance, however, varied around the distance of the target object used in the standard stimulus condition (middle of the display) from −4.5 cm (i.e., 4.5 cm closer to the observer) to +4.5 cm (i.e., 4.5 cm farther apart) in ten equidistant steps. Participants had to estimate whether the first (left mouse button) or the second (right mouse button) target object was farther away.

In the *size judgment blocks,* the distance of the target object was always the same (middle of the display). Its height, however, varied around the height of the target object used in the standard stimulus condition (4.3 × 3.1 cm) from −1.8 cm (i.e., 1.8 cm smaller than the standard stimulus) to +1.8 cm (larger than the standard stimulus) in ten equidistant steps. Participants had to estimate whether the first (left mouse button) or the second (right mouse button) target object was larger.

The experiment was divided into two separate sessions taking place on two different days and lasting about 1.2 h on average. Each session included four distance judgment blocks and four size judgement blocks. The order of blocks was DSDSDSDS (D = distance judgment block, S = size judgment block) for 15 participants and SDSDSDSD for fourteen participants in each of the sessions. One participant was exposed to each of these orders due to inattention of the experimenter.

Each block included 40 trials. In one half of the trials, the first target object was a standard stimulus and the second target object a test stimulus. For the other trials, the reverse was true. The order of these and all other conditions (2 movement distance conditions × 10 test stimuli) was random. Overall, participants performed 16 repetitions of each movement distance and test stimulus condition for each type of block. At the beginning of each session, participants performed eight practice trials for each block type, which were not included in the analysis.

#### Data analysis

The proportion of trials in which the test stimulus was judged as larger in the size judgment blocks and farther away in the distance judgment blocks was computed as a function of the test size and movement distance. Two participants were excluded from further analyses due to very low discrimination performance in the distance blocks (see participants 18 and 23 in Fig. S1 in the Supplementary Materials). A local model-free fitting procedure was applied to fit psychometric functions (Zychaluk & Foster, 2009). The point of subjective equality (PSE) was determined for each movement distance and type of judgment by estimating the test value at which the test stimulus was judged as larger/farther away with a frequency of 50%. The raw data have been made publicly available (https://osf.io/3unkb/).

We also quantified the predictions of the SDIH. That is, based on the impact of hand movement distance on the target distance judgments we predicted its effect on target size judgments. For this purpose, the perceived horizontal distance to the target’s center (a’) was transformed to the perceived eye-target distance (c’) according to Pythagoras's theorem for each participant and each movement distance condition (see also the right upper part of Fig. 1):

$$ {c}^{\hbox{'}}=\sqrt{{a^{\hbox{'}}}^2+{b}^2} $$

where *a*^'^ = *a* + *PSE*; $a=\sqrt{c^2-{b}^2}$; *b* = 325 *mm*; and *c* = 900 *mm*.

The to be perceived size of the target was then computed for each movement distance condition using the following formula (see, e.g., Kilpatrick & Ittelson, 1953):

$$ s\hbox{'}=\left(\frac{c^{\hbox{'}}\cdotp s/2}{c}\right)\cdotp 2 $$

where *s* = 43 *mm*.

#### Hypotheses

The PSE was expected to be larger for the large movement distance than for the small movement distance. This should be true for each type of judgment.

### Results and discussion

The PSE was larger for the large than for the small movement distance condition in the judgments of target distance, *t*(27) = 4.68, *p* < .001, as well as in the judgments of target size, *t*(27) = 3.32, *p* = .003, as predicted (see Fig. 2b–c for mean PSEs and the corresponding judgment data, and Fig. S1 in the Supplementary Materials for individual judgment data). These results suggest that the participants combined the proprioceptive with the visual cues in the estimation of target distance and that this multimodal percept of target distance affected the perceived size of the target object.

We also explored whether there is a correlation between both effects across the participants. After excluding two obvious outliers for this analysis (whose distance effect was more than 2.5 standard deviation larger than the mean) there was a trend towards a positive correlation, *r* = .339, *p* = .090, indicating that participants who showed a large effect in the distance blocks where those who also showed a large effect in the size blocks (see Fig. 2d).^1^ This is another indicator that the impact of proprioception on the distance perception propagated to the size perception. Participants with larger effects were likely those who weighted the proprioceptive information more strongly.

The SDIH predicted, however, a slightly smaller magnitude of the effect on size judgments (*M* = 0.3 mm, *SD* = 0.3) than we observed (*M* = 0.8 mm, *SD* = 1.2), *t*(27) = 2.0, *p* = .056. There may be at least two reasons for this trend. It is possible that our approximation to the SDIH and/or measurements did not capture the size constancy exactly. Also, since the impact of different cues can depend on task conditions (e.g., Cutting & Vishton, 1995) proprioception could have received slightly more weight in the size judgment blocks than in the distance judgment blocks (also see the General Discussion).

## Experiment 2

In Experiment 2, we used the same virtual reaching and grasping task as in Experiment 1, but now varied the gain of the grasping component of the hand movement (i.e., finger aperture). An increase in finger aperture was expected to increase the perceived size of the target (see also Kirsch et al., 2017; Kirsch & Kunde, 2019a, 2019b; for related observations). This should be so because the combined multimodal estimate of the target size should be smaller for the small than for the large finger aperture. Moreover, an increase in finger aperture could also increase the perceived distance of the target according to the SDIH. However, matters are complicated as the SDIH is often violated when size and distance judgments are concurrently collected in the same experiment and the apparent object’s size varies (Epstein et al., 1961). For example, the moon appears as larger at the horizon than at the zenith (the moon illusion), while it is concurrently judged as being closer at the horizon than at the zenith. This inconsistency, according to the SDIH, is called the size distance paradox (SDP; Epstein et al., 1961; Gruber, 1954; Kaufman et al., 2006; Ono et al., 1974). We were thus prepared to encounter both effect directions.

### Methods

#### Participants

The sample size was determined to be 32 participants prior to data collection. The sample included twenty-four females and eight males (*M*_age_ = 26 years, *SD* = 6 years). All participants were right-handed and had normal or corrected-to-normal vision. They gave their written informed consent for the procedures and received monetary compensation or course credit for their participation.

#### Apparatus

The apparatus was the same as in Experiment 1.

#### Stimuli and trial procedure

Stimuli and trial procedure were the same as in Experiment 1.

#### Design

The design was the same as in Experiment 1, except for the following change. The critical experimental variation was now related to the transformation of the distance between the fingers into the distance between the cursors. In the “small finger aperture” condition, the distance between the fingers was 1.1 cm smaller than the size of the target object (4.3 cm). In the “large finger aperture” condition, the distance between the finger was 1.1 cm larger than the size of the target object. This finger aperture variation was applied only for movements to the target object that served as a standard stimulus. Enclosing of the target object that served as a test stimulus was not transformed (i.e., the distances between the fingers corresponded to the size of the target object displayed on the monitor). Also, the reaching component of the movements was always the same (i.e., hand movement distance always amounted about 11 cm for the targets presented in the middle of the display; also see Experiment 1).

#### Data analysis

Data analysis was performed in the same way as in Experiment 1. The only difference was that the factor finger aperture was used in Experiment 2 instead of the factor movement distance used in Experiment 1. One participant had to be excluded due to very low discrimination performance in the size blocks (see Participant 24 in Fig. S2 in the Supplementary Materials).

We again quantified the predictions of the SDIH. Here, the impact of finger aperture on target distance judgments was predicted based on its effect on target size judgments. The perceived eye-target distance (c’) was initially computed for each finger aperture condition using the following formula (see e.g., Kilpatrick & Ittelson, 1953):

$$ c\hbox{'}=\left(\frac{c\cdotp s\hbox{'}/2}{s/2}\right) $$

where *s*^'^ = *s* + *PSE*; *s* = 43 *mm*; and *c* = 900 *mm*.

Then, the to be perceived horizontal target distance (a’) was derived for each finger aperture according to:

$$ {a}^{\hbox{'}}=\sqrt{{c^{\hbox{'}}}^2-{b}^2} $$

where *b* = 325 *mm*.

#### Hypotheses

The PSE of size judgments was expected to be larger for the large finger aperture than for the small finger aperture. For target distance judgments, the PSE should increase for the large finger aperture as compared with the small finger aperture according to the SDIH, but to decrease according to the SDP.

### Results and discussion

**Fig. 3:**
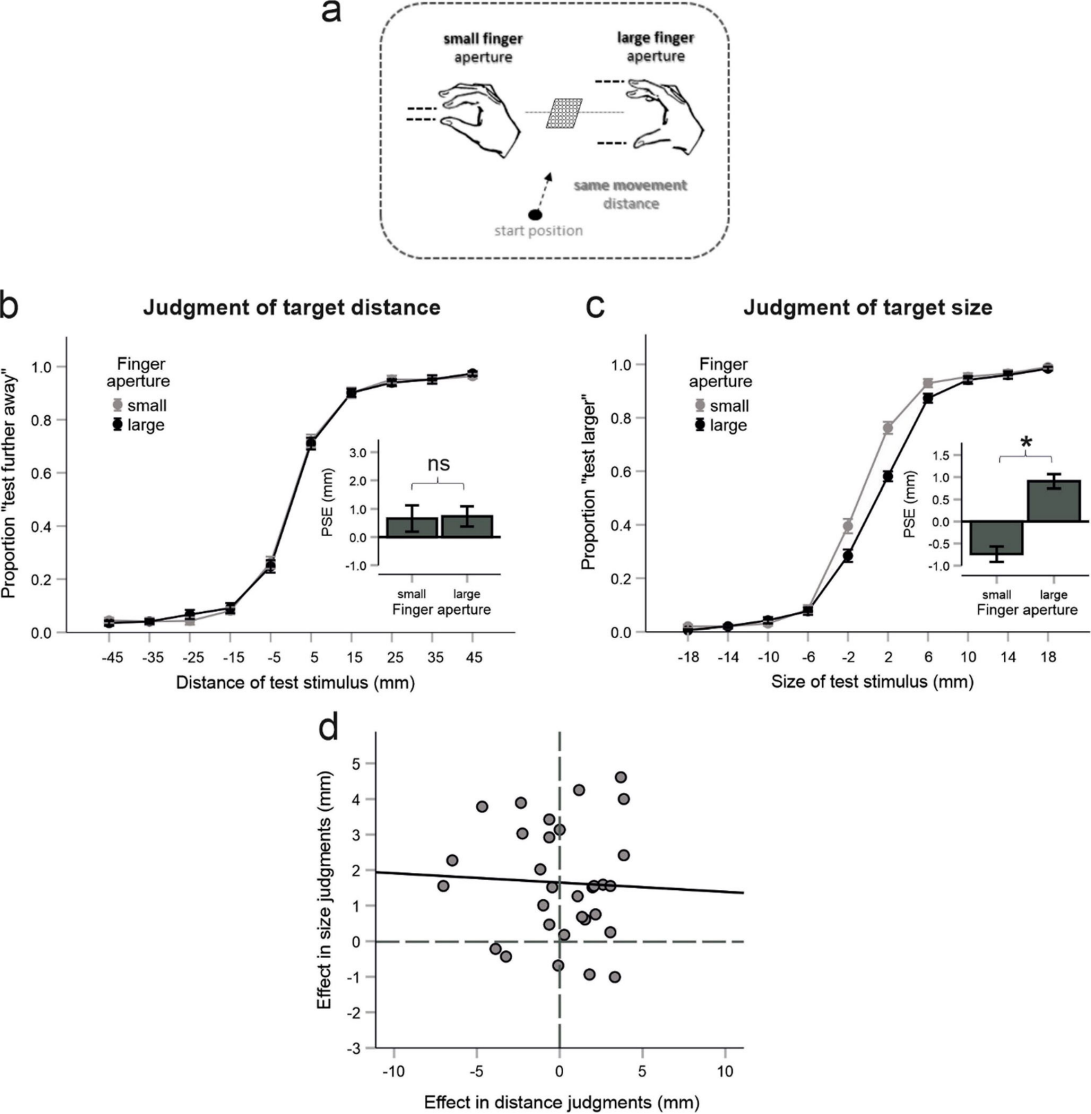
Experiment 2. **a** The critical variation of finger aperture. **b** Results for the judgments of target distance. Proportions of trials in which the test stimulus was judged as farther away as a function of finger aperture and the distance of the test stimulus. Negative/positive values mean that the test stimulus was closer/farther away than the standard stimulus. Superimposed are the mean PSE values for the large and small finger aperture conditions. **c** Results for the judgments of target size. Proportions of trials in which the test stimulus was judged as larger as a function of finger aperture and the size of the test stimulus. Negative/positive values mean that the test stimulus was smaller/larger than the standard stimulus. Superimposed are the mean PSE values for the large and small movement distance conditions. **d** The effect of finger aperture (large minus small) in the size judgments against the effect of finger aperture in the distance judgments for each participant. Error bars are standard errors indicating the variability across participants. Asterisk denotes statistical significance (*p* < .05)

The PSE was larger for the large than for the small finger aperture in the judgments of target size, *t*(30) = 5.73, *p* < .001, but there was no difference between the finger apertures in the judgments of target distance, *t*(30) = .15, *p* = .882 (see Fig. 3b–c for mean PSEs and the corresponding judgment data, and Fig. S2 in the Supplementary Materials for individual judgment data). These results suggest that the participants combined their finger aperture with the visual cues in the estimation of target size and that this multimodal percept of target size did not affect the perceived distance of the target object.

We again explored whether there is a correlation of the difference between finger apertures measured in the distance judgment blocks and that measured in the size blocks across the participants. There was no indication for such a relation, *r* = .048, *p* = .798 (see Fig. 3d). This is another indicator that the impact of proprioception on the size perception did not propagate to the distance perception.

The SDIH predicted a mean PSE difference of 36.9 mm (*SD* = 35.9) for the target distance judgments between both finger aperture conditions. This value was far above the observed difference (*M* = 0.1 mm, *SD* = 2.9), *t*(30) = 5.67, *p* < .001. Although we did not quantify the SDP explicitly, we also did not observed evidence for the SDP as there were no signs of an inverse relation between perceived size and distance. Thus, neither the SDIH nor the SDP were supported by the results.

## General discussion

The present study explored the origin of action specific effects in visual perception. Based on a sensory integration approach we hypothesized that the perception of distant objects in the context of actions is directly affected via exploitation and integration of redundant multimodal signals and tested whether these direct influences can lead to indirect perceptual effects via size constancy mechanisms. In a virtual reaching and grasping task, we introduced a crossmodal conflict between visual object features and proprioceptive object information provided by the reaching (Experiment 1) or grasping (Experiment 2) part of the action and measured the effects of these manipulations on the perception of a target object.

An increase in proprioceptive object distance increased the perceived object distance in Experiment 1, whereas an increase in proprioceptive object size increased the perceived object size in Experiment 2. These results are in accordance with direct effects of sensory integration on the perception of distant objects. In addition, an increase in proprioceptive object distance also increased the perceived object size. This outcome suggests an indirect influence of sensory integration on perception via size constancy mechanisms. An increase in proprioceptive object size, however, did not affect the perceived object distance.

There may be several reasons for why changes in size perception did not propagate to the changes in distance perception. One idea stems from the research on the SDP that is often explained by a two-stage mechanism (e.g., Coren & Aks, 1990; Higashiyama, 1979). Initially, distance cues are used to scale the retinal image size and thus determine perceived object’s size in accord with the SDIH. At this stage, if distance cues signal a larger distance an object (of constant retinal size) is perceived as larger. Then, perceived size is used as a cue for judgments of object’s distance while distance cues are ignored. At this second stage, apparently larger objects, which were associated with a larger distance initially, are now judged as closer. This presumably occurs because the physical distance usually increases when the retinal objects’ size decreases. Accordingly, it has been argued that such distance judgments that violate the SDIH reflect participants’ cognitive responses (i.e., a response bias), rather than being perceptual in nature (Kaufman et al., 2006; Kaufman et al., 2007; Mon-Williams & Tresilian, 1999; see also Gogel & Sturm, 1971; Ono et al., 1974, for similar suggestions). This approach indicates that distance cues used for size and distance judgments do not need to be the same and that distance perception does not necessarily rely on perceived size. In the present study, we combined a two-alternative forced-choice procedure with the method of constant stimuli. This approach is rather immune to response biases (see also Kaufman et al., 2007). Thus, assuming that size constancy mechanisms hold for the perceived size but not for the perceived distance and that we measured genuine perception would explain why an impact of perceived size (affected by proprioception) on perceived distance was not observed.

Alternatively, it is also possible that the size constancy holds for both perceived distance and size. However, the introduced conflict between hand opening and visual/ocular signals could have been treated differently for the perception of size and distance. This conflict could be tolerated in size perception, but perhaps not in distance perception. In particular, while proprioceptive information received some weight in the integrated percept of size, it could have been received no weight at all in the perception of distance (see, e.g., Brenner & van Damme, 1999). This could be because relatively small errors in apparent size could cause relatively large errors in distance perception (see also the SDIH predictions for Experiment 1 and Experiment 2). This would be in accordance with a common assumption that sensory cues are used and weighted according to given task conditions (e.g., Cutting & Vishton, 1995; Landy et al., 1995).

Previous studies manipulating action related variables revealed effects on both, size and distance perception (see also the Introduction). The present results suggest that these observations might be of a similar origin, at least to a certain degree. In particular, using tools extending observers’ reachability proved to decrease the perceived distance to a distant target object (e.g., Linkenauger et al., 2015; Witt, 2011b; see also Introduction). This effect should be accompanied by an increase in perceived object size given the present results. Such an effect has in fact been reported (Suh & Abrams, 2018). Moreover, in many sports-like tasks, such as archery, in which action ability affected size perception, one might wonder how an action such as shooting an arrow, whose obvious purpose is to bridge a certain distance, can affect the perceived size of an object (Lee et al., 2012; see also the Introduction). One possibility indicated by the present results is that in such situations action related variables primarily affect distance perception and that the effects on perceived size are a by-product of the size constancy. These considerations are of course tentative and should be considered with caution. In theory, two originally unrelated arbitrary signals can be integrated after a systematic statistical relation between them is established (Ernst, 2007; Kaliuzhna et al., 2015). Accordingly, in principle, any sensory features of an action can be assumed to be directly linked to any visual characteristics of an object based on their statistical co-occurrence.

Perceptual effects observed in the context of actions have been often discussed within two theoretical frameworks. First, action-related variables, such as ability, have been suggested to scale early sensory processing (e.g., Proffitt & Linkenauger, 2013; Witt, 2011a). Changing these variables—that is, the reference scale—is assumed to change the percept. Second, it has been proposed that actions and perceptions are commonly coded in cognition (e.g., Hommel et al., 2001). Here, perceptual changes can emerge when features of motor and perceptual codes overlap. The present results do not contradict these theories. However, specific links to size constancy in general and to the present and related task situations in particular cannot be inferred from these ideas, at least not without additional assumptions. The sensory integration approach, in contrast, puts action related effects into context of a broad traditional research on perception and thus allows predictions that are more specific.

To conclude, the present results indicate that perceptual effects observed in the context of actions can be a direct consequence of sensory integration of bodily and visual signals relating to the same feature of an object. However, such effects can propagate to a different object feature through perceptual constancy mechanisms. In that case, a certain body-related characteristic of an object can indirectly affect a different object characteristic.

## Supplementary Information

ESM 1 (DOCX 848 kb)

## Footnotes

1 Excluding these participants from the main analyses did not change the results substantially. The PSE was still significantly larger for the small movement distance in the distance judgment blocks, *t*(25) = 8.71, *p* < .001, as well as in the size blocks, *t*(25) = 4.10, *p* < .001.
