# Supplementary material for: Impact of proprioception on the perceived size and distance of external objects in a virtual action task
Source: Psychon Bull Rev. 2021 Mar 29;28(4):1191–201. doi: 10.3758/s13423-021-01915-y (PMC8367880; doi:10.3758/s13423-021-01915-y)
Supplement: Supplementary file 1 — (DOCX 848 kb) [file 13423_2021_1915_MOESM1_ESM.docx]

**Supplementary materials**

***
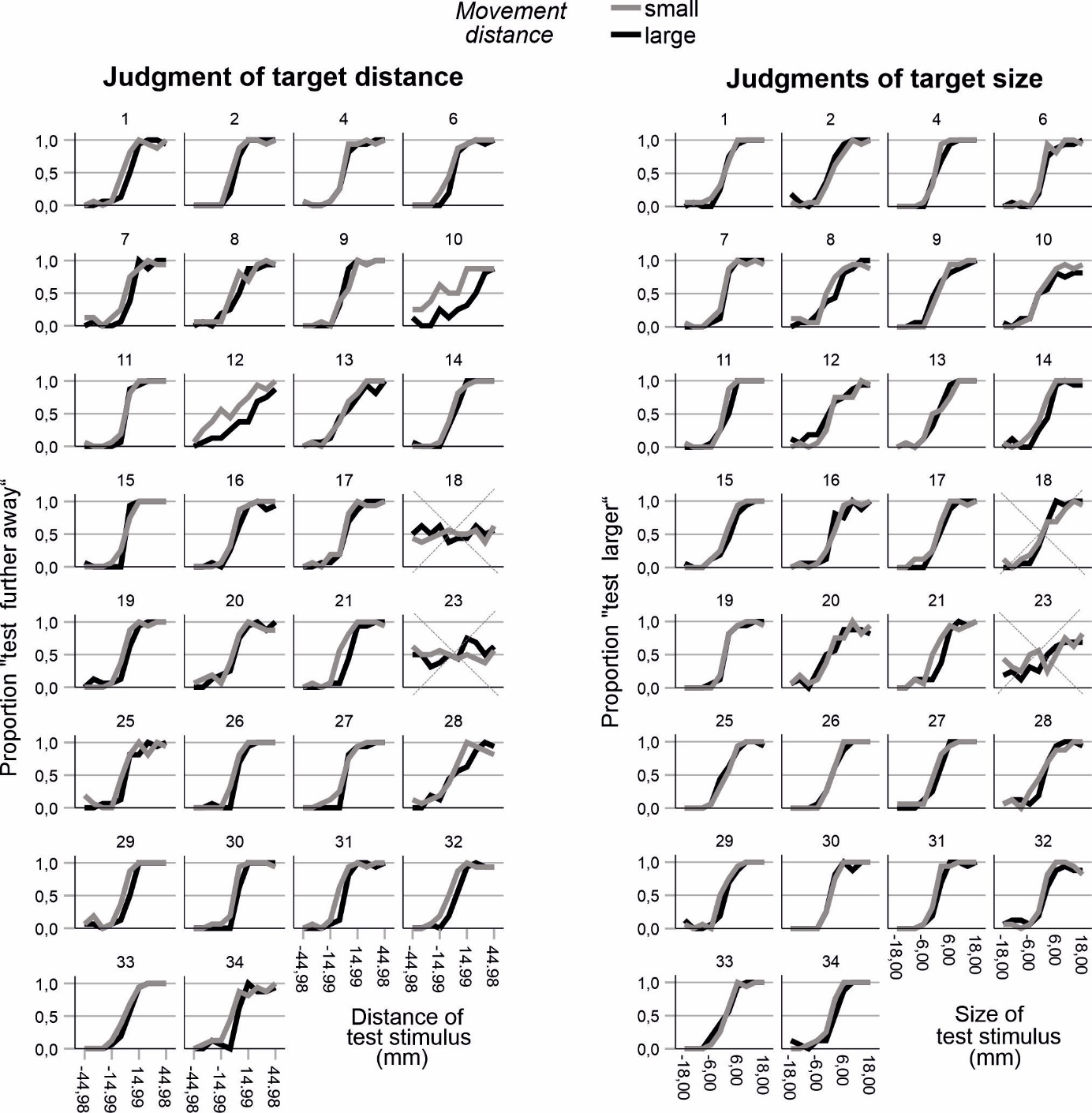
***

***Figure S1.*** *Individual judgment data for Exp.1. Values indicate the proportion of trials in which the test stimulus was judged as further away (distance judgment) / larger (size judgment) as a function of movement distance and the distance (distance judgment) / size (size judgment) of the test stimulus. Gray crosses indicate participants with low discrimination performance (in at least one type of blocks) who were not included in the analyses.*

***
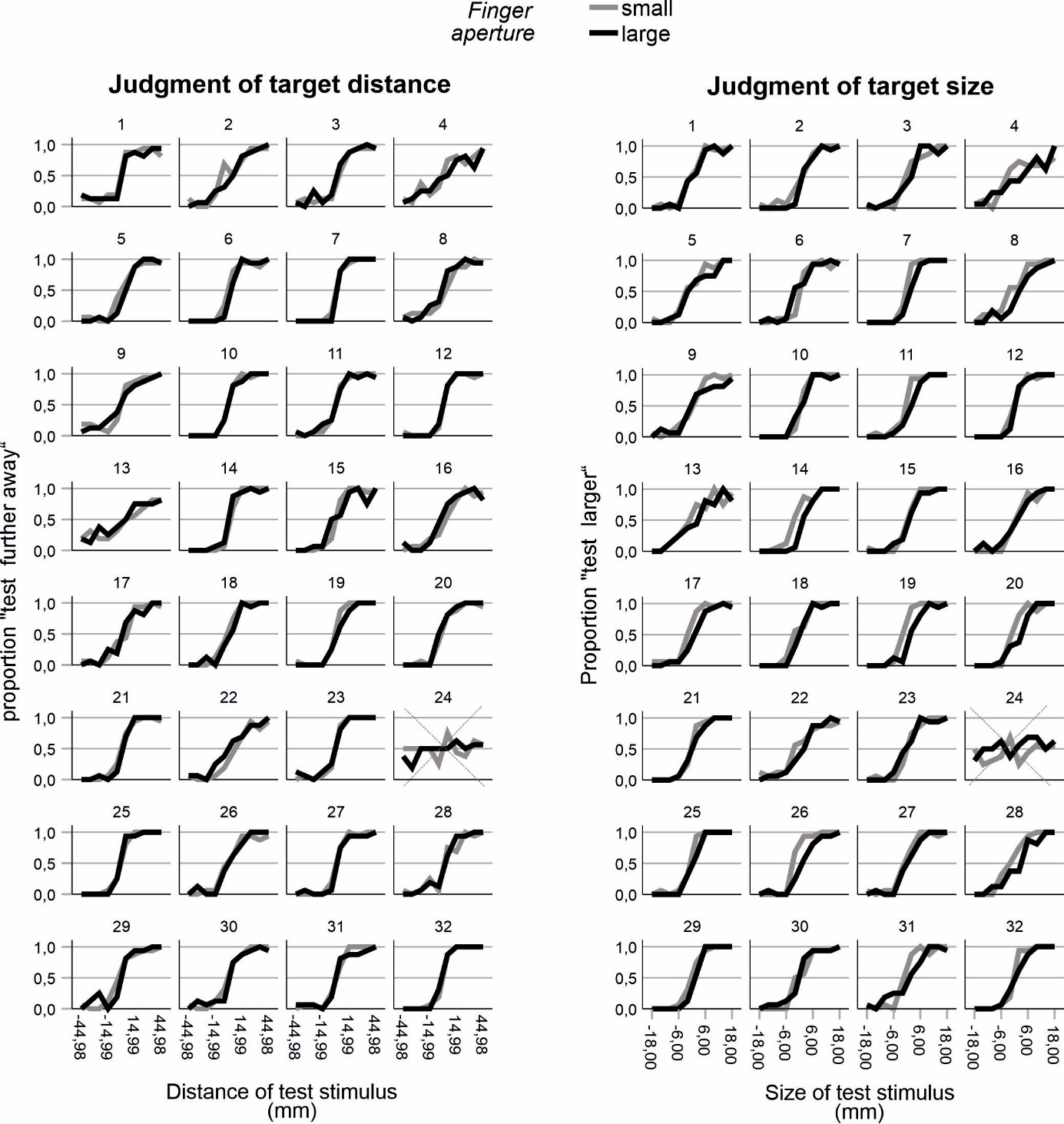
Figure S2.*** *Individual judgment data for Exp.2. Values indicate the proportion of trials in which the test stimulus was judged as larger (size judgment) / further away (distance judgment) as a function of finger aperture and the size (size judgment) / distance (distance judgment) of the test stimulus. Gray cross indicates a participant with low discrimination performance who was not included in the analyses.*
